# Cancer-Associated Retinopathy Presenting with Paraneoplastic Cerebellar Degeneration in Tonsillar Squamous Cell Carcinoma: A Case Report

**DOI:** 10.3390/reports9030307

**Published:** 2026-09-11

**Authors:** Yeseo Kwon, Pooja Raibagkar, Bharat B. Yarlagadda, Jayashri Srinivasan, David J. Ramsey

**Affiliations:** 1 Division of Ophthalmology, Department of Surgery, UMass Chan-Lahey School of Medicine, Burlington, MA 01805, USA; yeseo.kwon@tufts.edu; 2 Department of Ophthalmology, Tufts University School of Medicine, Boston, MA 02116, USA; 3 Department of Neurology, UMass Chan-Lahey School of Medicine, Burlington, MA 01805, USA; 4 Division of Otolaryngology-Head and Neck Surgery, Department of Surgery, UMass Chan-Lahey School of Medicine, Burlington, MA 01805, USA; 5 Department of Neurology, Tufts University School of Medicine, Boston, MA 02116, USA

**Keywords:** paraneoplastic syndromes, ocular, paraneoplastic cerebellar degeneration, tonsillar neoplasms, autoimmune diseases, retinal diseases, case reports

## Abstract

**Background and Clinical Significance:** Cancer-associated retinopathy (CAR) is a rare paraneoplastic autoimmune retinopathy caused by antibodies targeting tumor antigens with cross-reactivity to retinal proteins. Paraneoplastic cerebellar degeneration (PCD) results from autoimmune injury to cerebellar tissue triggered by onconeural antigens. We report a rare case of CAR occurring concurrently with seronegative PCD in a patient with poorly differentiated tonsillar squamous cell carcinoma, and we describe the long-term sequelae of CAR-related macular atrophy; **Case Presentation:** A 66-year-old male presented with progressive painless bilateral peripheral and night vision loss, truncal ataxia, dysmetria, and dysarthria. Optical coherence tomography demonstrated diffuse outer retinal loss, and multifocal electroretinography showed markedly reduced responses bilaterally. Evaluation revealed poorly differentiated squamous cell carcinoma of the right tonsil. CAR antibody testing was positive for recoverin, enolase, and carbonic anhydrase II antibodies. Brain MRI demonstrated cerebellar atrophy. After exclusion of other autoimmune etiologies, he was diagnosed with seronegative PCD. Treatment included robotic tonsillectomy with neck dissection, intravenous immunoglobulin, high-dose corticosteroids, and maintenance rituximab. At 6 months, visual acuity, color vision, and electro-retinography improved. Seven years later, he remains independent in activities of daily living despite the progression of retinal atrophy and cerebellar dysfunction; **Conclusions:** This case highlights the rare coexistence of CAR and seronegative PCD associated with tonsillar squamous cell carcinoma. Multimodal oncologic and immunosuppressive therapy produced meaningful neurologic and visual improvement, despite progressive long-term retinal degeneration.

## 1. Introduction and Clinical Significance

Cancer-associated retinopathy (CAR) is a rare paraneoplastic syndrome characterized by retinal dysfunction resulting from antibodies generated against tumor antigens that cross-react with retinal proteins, leading to photoreceptor degeneration and vision loss [1]. CAR is a form of autoimmune retinopathy, often presenting with sudden onset of photopsias, painless rapid visual loss, and abnormal electroretinogram (ERG) findings [1]. Several antiretinal antibodies have been implicated, including recoverin, α-enolase, tubby-like protein 1 (TULP1), heat shock cognate protein 70 (HSC70), glyceraldehyde-3-phosphate dehydrogenase, and carbonic anhydrase II (CAII), although clinical manifestations vary according to the underlying antibody profile [1]. CAR is most commonly associated with lung, breast, gynecologic, prostate, and colorectal malignancies, as well as cutaneous melanoma and hematologic cancers [1].

Similarly, paraneoplastic cerebellar degeneration (PCD) is an immune-mediated neurologic syndrome characterized by rapidly progressive pancerebellar dysfunction caused by antibodies directed against onconeural antigens expressed by both tumor cells and cerebellar tissue [2]. The most frequently identified antibodies include anti-Yo (PCA-1; Purkinje cell cytoplasmic antibody type 1), anti-Hu (ANNA-1; antineuronal nuclear antibody type 1), anti-voltage-gated calcium channel (VGCC), anti-Ri (ANNA-2; antineuronal nuclear antibody type 2), anti-CV2/CRMP5 (collapsin response mediator protein 5), anti-Tr/DNER (Delta/Notch-like epidermal growth factor-related receptor), anti-mGluR1 (metabotropic glutamate receptor 1), anti-KLHL11 (Kelch-like protein 11), and anti-MAP1B (microtubule-associated protein 1B); of these, anti-Yo and anti-Hu are the most frequently identified [3]. Notably, none of the antibodies linked to PCD overlap with those associated with CAR. However, seronegative presentations of both CAR and PCD complicate identification of the underlying mechanism in a substantial number of cases. Among patients with symptoms of CAR, up to a third are seronegative for known antibodies implicated in this complex autoimmune condition [1]. Similarly, seronegative PCD accounts for approximately 18% of all patients with the condition, with gynecological cancers and lymphomas predominating in women and lung and genitourinary cancers in men [4].

Here, we describe a case of CAR with antibodies identified against recoverin, enolase, and CAII in a patient who simultaneously manifested cerebellar ataxia and degeneration in the setting of poorly differentiated squamous cell carcinoma of tonsil, a malignancy not previously linked to CAR. While potential overlap between these anti-retinal antibodies and PCD has not been previously described, given the wide range of aberrant proteins expressed by poorly differentiated oncologic cells, and taking into account the previously described concept that a single autoimmune process may target both cerebellum and retina, a yet unidentified variant or cross-reactive target may allow for potential overlap of CAR and PCD [2]. Additionally, the patient’s 7-year follow-up provides a rare opportunity to gain insight into the long-term retinal sequalae of CAR.

## 2. Case Presentation

**Figure 1:**
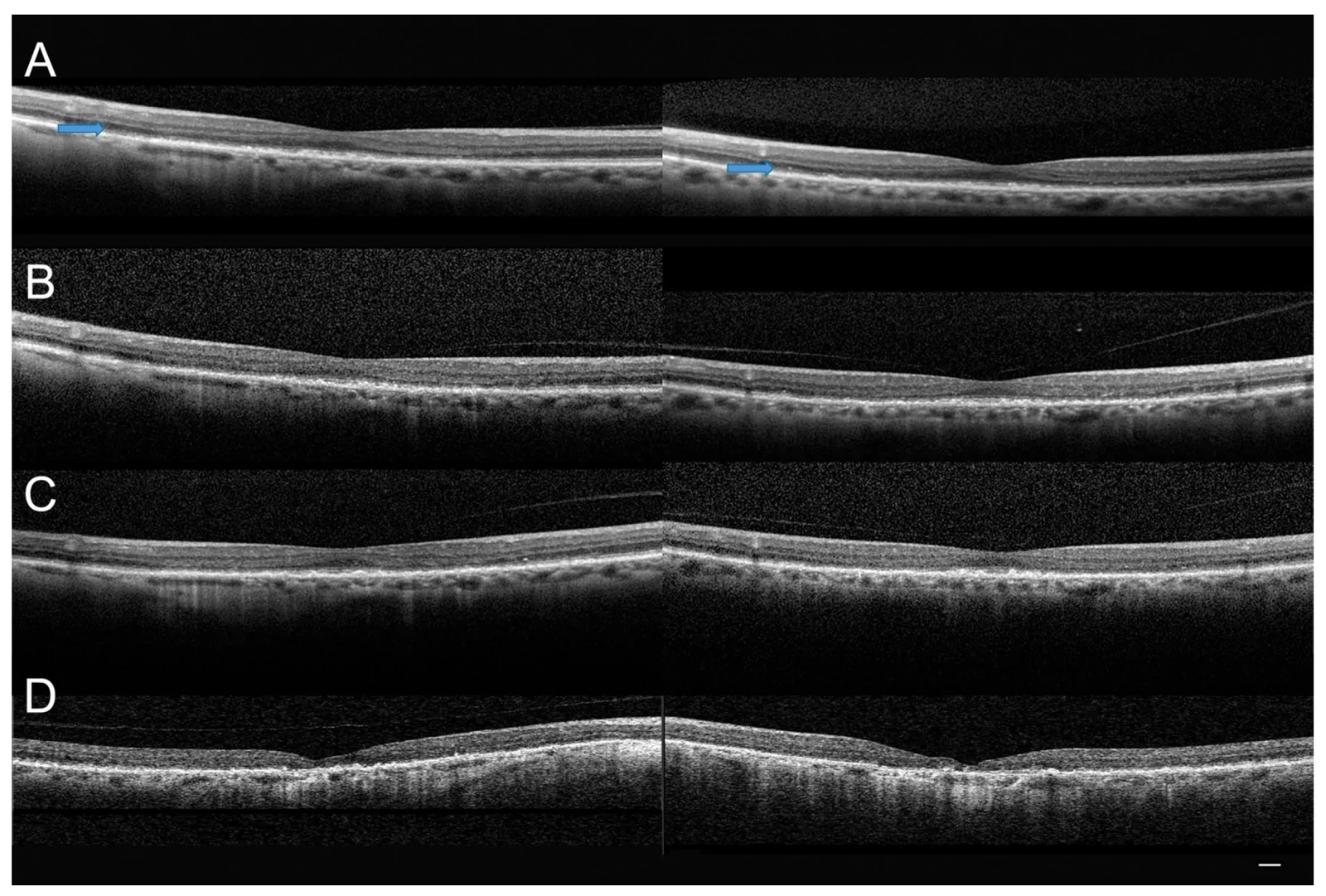
OCT scans of the macular of the right eye and left eyes at presentation (**A**), at seven months after immunotherapy treatment (**B**), and at one year (**C**). The images obtained at presentation show marked retinal thinning with loss primarily in the outer retinal layers, including the outer nuclear and photoreceptor layers. Disruption of the ellipsoid zone (inner segment/outer segment junction; blue arrows) was evident at presentation but improved following treatment. At most recent follow-up seven years later, OCT scans of the right and left eyes (**D**) continue to show marked thinning of the neurosensory retina with reverse shadowing consistent with subfoveal RPE atrophy in both eyes, which accounts for the decline in visual acuity. Scale bar 200 µm.

**Figure 2:**
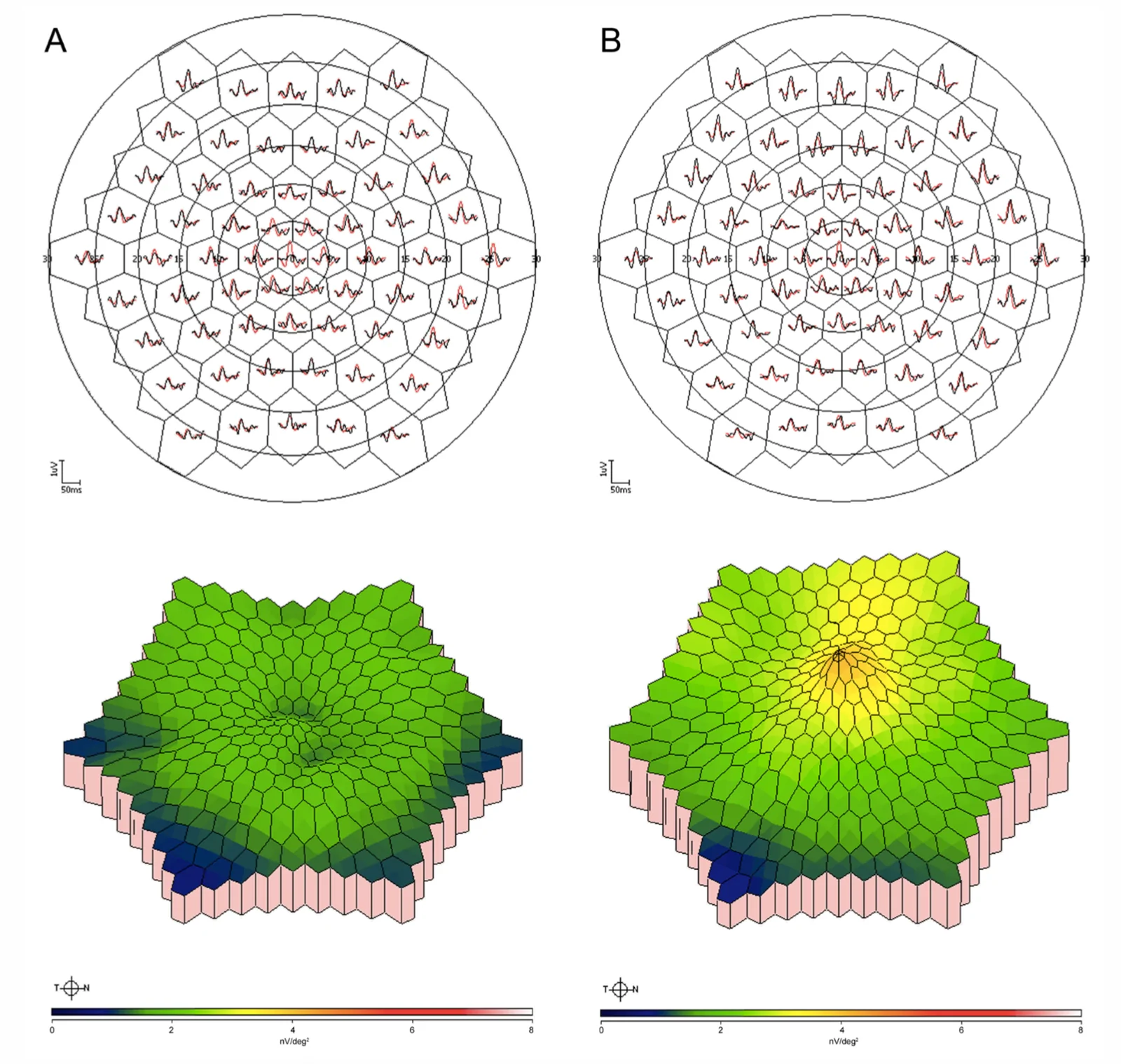
The mfERG illustrates reduced amplitude with increased latency in the right eye at presentation (**A**) with improvement in amplitudes and waveforms after immunotherapy treatment (**B**). Less pronounced improvement was noted in the left eye. Top: The mfERG display showing 61-scaled hexagons with circles drawn to indicate radii in 5° increments up to 30°. Within each hexagon is the response (first-order kernel) extracted by correlating the stimulus sequence with the continuous ERG record. Bottom: The 3D mfERG density plot of the responses.

A 66-year-old male presented with a 6-month history of progressive, painless peripheral and night vision loss, more pronounced in the right eye, accompanied by a 2-month history of worsening gait instability and dysarthria (Supplemental Figure S1). Neurologic examination demonstrated dysmetria involving all extremities, greater on the left; severe truncal ataxia; brisk patellar reflexes with diminished Achilles reflexes bilaterally; and length-dependent loss of vibration and temperature sensation below the mid-shin. Cranial nerve, motor, and mental status examinations were otherwise normal. He required two-person assistance to ambulate. On ophthalmologic examination, best-corrected visual acuity (BCVA) was 20/50 OD and 20/60 OS. Color vision was reduced bilaterally (Ishihara score 2.5/10 OD and 3.5/10 OS). Spectral-domain optical coherence tomography (OCT) showed diffuse outer retinal loss (Figure 1A). While the full-field electroretinogram (ffERG) was largely intact, the multifocal electroretinogram (mfERG) was diffusely depressed in both eyes (Figure 2A). Dark adaptometry (DA) revealed severely impaired rod-mediated responses, with no measurable rod-intercept time in either eye (AdaptDx, Maculogix Inc., Harrisburg, PA, USA).

Brain magnetic resonance imaging (MRI) showed cerebellar atrophy. Cerebrospinal fluid (CSF) analysis showed elevated nucleated cells 54 cells/µL (70% Neutrophils, 15% lymphocytes) and elevated protein 57 mg/dL (normal 15–45 mg/dL). Cervical spinal MRI showed spinal cord compression from large disc protrusion at C4–C5 and myelomalacia. Extensive evaluation for autoimmune and degenerative causes of cerebellar ataxia, including comprehensive serum and CSF paraneoplastic antibody panels, was unrevealing (antineuronal nuclear antibodies (ANNA-1, ANNA-2, ANNA-3), anti-glial nuclear antibody-1 (AGNA-1), Purkinje cell antibodies (PCA-1, PCA-2, PCA-TR), amphiphysin ab, collapsing response mediator protein-5 (CRMP-5-IgG), striational ab, P/Q type calcium channel ab, N-type calcium channel ab, acetylcholine receptor (AChR) binding ab, AChR ganglionic neuronal ab, neuronal (V-G) K+ channel ab, glutamic acid decarboxylase-65 (GAD65), zic4 ab, metabotropic glutamate receptor (mGluR1), dipeptidyl-peptidase-like protein 6 (DPPX ab), adaptor-related protein complex 3 subunit beta 2 (AP3B2) ab, and homer-3 in serum). Screening for autoimmune and degenerative causes of cerebellar ataxia including vitamin E deficiency, multi-system atrophy, hypothyroidism, and multiple myeloma was unrevealing. By contrast, a CAR immunohistochemistry panel showed elevated titers of recoverin, enolase and CAII antibodies (Mayo Clinic Laboratories, Rochester, MN, USA). An inherited retinal dystrophy (IRD) panel examining 363 genes demonstrated no sequence variants likely to be contributory (PreventionGenetics LLC, Marshfield, WI, USA).

While undergoing endotracheal intubation for C4-C5 anterior cervical discectomy and fusion for cervical stenosis, the patient was incidentally found to have large pedunculated right tonsil mass measuring 2.5 cm. Biopsy demonstrated poorly differentiated squamous cell carcinoma (clinical stage T2). Over the subsequent two months, BCVA declined to 20/150 OS and subsequently to 20/200 OS, despite no evidence of recurrent or metastatic disease on staging evaluation. Dark adaptation threshold and time was elevated in both eyes with prolonged rod/cone break point. The patient underwent a robotic right tonsillectomy with neck dissection. Because of favorable pathologic features, including an absence of nodal involvement, no adjuvant therapy was recommended. Follow-up imaging did not reveal any recurrent or metastatic disease.

The patient was treated with intravenous immunoglobulin (IVIG) and high-dose corticosteroids for presumed paraneoplastic retinal and cerebellar disease. Six months after treatment, BCVA improved to 20/25-2 OD and 20/60 + 2 OS; Ishihara color vision improved to 6/10 in each eye, mfERG responses improved OU (Figure 2B), and outer retinal architecture partially recovered on OCT imaging (Figure 1B). Neurologically, gait improved to the point that the patient was able to ambulate with a walker. Following this initial course of immunotherapy, the patient continued to report significant clinical and functional improvement. At one-year follow-up, visual acuity, color vision, and mfERG responses remained improved, and outer retinal and photoreceptor architecture on OCT imaging continued to show partial restoration of the photoreceptor ellipsoid zone (Figure 1C), prompting an additional course of IVIG. Rituximab therapy, an anti-CD20 monoclonal antibody, at a dose of 1000 mg was initiated in the following year as a maintenance therapy to target T-cell cytotoxicity by decreasing antigen-presenting of B cells. This was followed by six additional courses of IVIG over the following year, two rituximab infusions two years later, and two additional infusions three years after that. In total, the patient received eight IVIG treatment cycles and five rituximab infusions.

**Figure 3:**
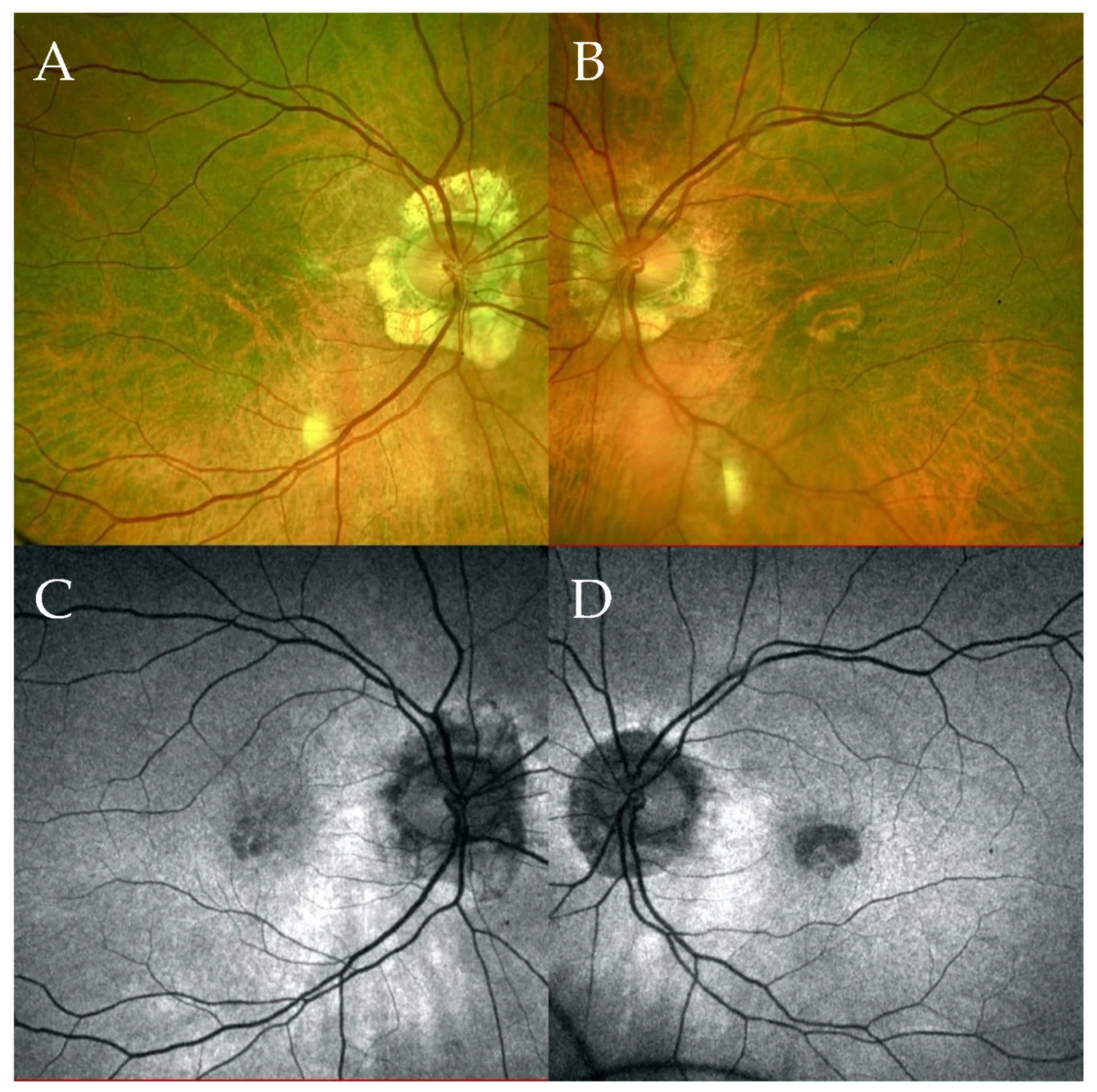
Fundus photographs and AF images of the right (**A**) and left (**B**) eyes six years after presentation demonstrate central macular atrophy in the left greater than the right eyes. Corresponding fundus autofluorescence imaging of the right (**C**) and left (**D**) eyes illustrate the loss of RPE autofluorescence involving the fovea of both eyes.

Five years after the patient’s initial visit, BCVA remained stable at 20/25 OD and 20/50 OS with sustained improvements in activity and gait. However, six years after presentation, visual acuity precipitously declined, first in the right eye (20/200), then shortly later in the left eye (20/250). Examination of the fundus demonstrated thinning of the neurosensory retinal with retinal pigment epithelium (RPE) atrophy involving the fovea of both eyes (Figure 3), which was confirmed by OCT imaging (Figure 1D). Low-vision rehabilitation did not improve visual function.

## 3. Discussion

We present, to our knowledge, the first reported case of CAR occurring concurrently with seronegative PCD in association with poorly differentiated tonsillar squamous cell carcinoma. The patient demonstrated antibodies against recoverin, enolase, and CAII. While extensive evaluation failed to identify a recognized PCD-associated antibody, the combination of characteristic cerebellar findings, exclusion of alternative etiologies, and an underlying malignancy strongly supported the diagnosis of seronegative PCD.

Recoverin, a calcium-binding protein found in photoreceptors, is often aberrantly expressed in tumor cells [1]. This results in anti-tumor antibody cross-reactivity with retinal cells which may lead to their apoptosis and an associated acute, severe vision loss [1]. Similarly, enolase, a ubiquitously expressed 46 kDa glycolytic enzyme found in many tissues, can induce cross-reactivity with forms of the protein found in photoreceptor cells and is associated primarily with cone dysfunction, which brings about a slowly progressing central vision loss [1]. While CAII antibodies have been implicated in many paraneoplastic retinopathies, the mechanism of cellular damage remains unclear. However, inhibition of CAII catalytic activity has been associated with increased intracellular calcium and decreased retinal cell viability [1]. The coexistence of multiple antiretinal antibodies in our patient likely contributed to the severe retinal phenotype.

Ataxia caused by PCD is exceedingly rare and has not been previously associated with CAR. Although PCD is classically associated with antibodies such as anti-Yo (PCA-1), anti-Hu (ANNA-1), anti-Ri (ANNA-2), anti-Tr/DNER, anti-CV2/CRMP5, and anti-mGluR1, approximately 18% of patients lack identifiable antibodies, despite a characteristic clinical syndrome [2,4]. While paraneoplastic ataxia has been described for various germ cell cancers, small-cell carcinomas, adenocarcinomas, and lymphomas, only five cases involving the tonsils have been described [3]. The cases, however, involved a lymphoepithelial carcinoma with anti-protein kinase C gamma (anti-PKCγ) antibodies in one of the cases, and others did not have identifiable antibodies that resulted in cerebellar degeneration with no retinal involvement [4,5]. Although exceedingly rare, squamous cell carcinoma of the tongue has been shown in one case to cause PCD with association to anti-CV2/CRMP5 antibodies but no retinal involvement [6]. Similarly, while recoverin is commonly associated with retinopathy, the only known case of recoverin antibody-induced ataxia did not involve retinopathy or neoplasia [7].

Inherited retinal diseases (IRDs), a diverse group of progressive, often blinding, genetic diseases, offer another potential cause included in the differential for vision loss alongside cerebellar ataxia [8]. The ophthalmologic symptoms in IRDs may appear similar to CAR, with loss of night vision, visual field, color, and central acuity, as certain IRDs can target rods and cones specifically [1,8]. Inherited neurodegenerative diseases such as spinocerebellar ataxia can demonstrate truncal ataxia as well as degeneration of retinal ganglion cells and progressive vision loss [9]. However, spinocerebellar ataxia typically presents from early to middle age and involves more gradual vision loss associated with nystagmus, slow saccades and/or ophthalmoplegia, which are typically absent in CAR where the vision loss is more acute in onset [1,9]. IRDs may also be associated with mitochondrial dysfunction, as opposed to the autoimmune pathology seen in CAR or PCD triggered by an underlying carcinoma [9]. Most importantly, IRDs can be identified with underlying genetic abnormality, with over 300 disease-causing genes currently characterized [10]. The patient’s late onset, rapidly progressive symptoms, positive antiretinal antibodies, concurrent malignancy, and negative 363-gene inherited retinal disease panel strongly favored a paraneoplastic, autoimmune process as opposed to an inherited neurodegenerative disorder.

The mechanism linking retinal and cerebellar injury remains uncertain. No antibody has been definitively shown to target both tissues in CAR and PCD. Nevertheless, experimental studies have demonstrated expression of retinal S-antigen (arrestin) and related proteins within the cerebellum, choroid plexus, and cerebrospinal fluid, raising the possibility of a shared antigenic target [11,12]. While not directly related to retinal degeneration, anti-CV2/CRMP5 antibodies associated with squamous cell carcinoma of the tongue in the aforementioned case of PCD may have played a role in vision loss, although the mechanism is distinct from CAR and melanoma-associated retinopathy (MAR) per se, in the form of optic disk edema, vitritis, and retinitis [6]. Although this hypothesis remains unproven, our case raises the possibility that unidentified cross-reactive antigens or immune mechanisms may contribute to simultaneous retinal and cerebellar injury in certain patients.

Treatment of paraneoplastic retinopathy and PCD remains challenging, as there are no established evidence-based treatment protocols for either condition. The primary therapeutic objective in paraneoplastic syndromes is the identification and treatment of the underlying malignancy, which may reduce the antigenic stimulus driving the immune response. For CAR, systemic corticosteroids are commonly used as first-line immunomodulatory therapy, with IVIG and plasma exchange also reported, particularly when there is rapid or progressive visual dysfunction [1,13]. Additional steroid-sparing or second-line immunosuppressive therapies, including rituximab, mycophenolate mofetil, and cyclophosphamide, have been described with variable efficacy. Although evidence is limited to case reports and small series, earlier treatment appears more likely to preserve residual retinal function before irreversible photoreceptor loss occurs [1,14,15]. In PCD, treatment similarly centers on prompt management of the underlying malignancy and early immunotherapy, most commonly corticosteroids, IVIG, or plasma exchange, with rituximab or other immunosuppressive agents considered for persistent or progressive disease. However, treatment responses are generally limited once irreversible Purkinje cell neuronal loss and cerebellar atrophy have developed [2].

An additional strength of this report is the prolonged clinical follow-up. Despite meaningful improvement in visual function, electroretinographic responses, and gait following tumor resection and immunotherapy, progressive bilateral macular RPE atrophy developed approximately six to seven years after diagnosis, resulting in substantial decline in vision with both eyes affected. This suggests that immunotherapy may have suppressed the active immune-mediated component without completely preventing downstream photoreceptor and RPE degeneration. This is consistent with the concept that autoimmune retinal injury may produce irreversible cellular damage despite subsequent control of the immune process [15]. Importantly, there was no temporal evidence in this case to establish IVIG or rituximab as a cause of the late-onset macular atrophy. Rather, the atrophy developed years after an initially positive response to immunotherapy and was accompanied by progressive thinning of the neurosensory retina with subfoveal RPE atrophy. Although age-related macular degeneration with geographic atrophy is a consideration given the patient’s age, the history of antibody-confirmed CAR, lack of drusen deposits, and no family history of similar age-relate eye disease make this diagnosis less likely [16]. Because many patients with CAR experience limited survival from their underlying malignancy, the long-term structural evolution of CAR is infrequently documented. Although an age-related contribution to the development of macular atrophy cannot be excluded, our findings suggest that the delayed bilateral macular RPE atrophy may represent a late structural sequela of CAR following early functional recovery.

## 4. Conclusions

We report, to our knowledge, the first case of CAR occurring concurrently with seronegative PCD in association with poorly differentiated tonsillar squamous cell carcinoma. This case expands the spectrum of malignancies associated with CAR, and it highlights that concurrent retinal and cerebellar paraneoplastic syndromes may occur despite the absence of identifiable PCD-associated antibodies. The patient’s favorable early response to tumor resection and immunosuppressive therapy establishes the importance of prompt recognition, followed by evaluation for an underlying malignancy, and early treatment in patients presenting with subacute visual loss and cerebellar ataxia. Finally, the seven-year follow-up provides rare insight into the long-term natural history of CAR and demonstrates that progressive macular RPE atrophy may develop, despite initial functional recovery.

## Data Availability

Supporting data can be found and is referenced within the publication. No other new data were created.

## Supplementary Materials

The following supporting information can be downloaded at: https://www.mdpi.com/article/10.3390/reports9030307/s1, Figure S1: Historical and current information from this episode of care organized as (A) neurologic and (B) ophthalmologic timelines.

## Abbreviations

The following abbreviations are used in this manuscript:

ANNA-1	Antineuronal nuclear antibody type 1
ANNA-2	Antineuronal nuclear antibody type 2
BCVA	Best-corrected visual acuity
CAII	Carbonic anhydrase II
CAR	Cancer-associated retinopathy
CRMP5	Collapsin response mediator protein 5
CSF	Cerebrospinal fluid
DNER	Delta/Notch-like epidermal growth factor-related receptor
ffERG	Full-field electroretinogram
IRD	Inherited retinal dystrophy
IVIG	Intravenous immunoglobulin
KLHL11	Kelch-like protein 11
MAP1B	Microtubule-associated protein 1B
MAR	Melanoma-associated retinopathy
mfERG	Multifocal electroretinogram
mGluR1	Metabotropic glutamate receptor 1
MRI	Magnetic resonance imaging
OCT	Optical coherence tomography
PCA-1	Purkinje cell cytoplasmic antibody type 1
PCD	Paraneoplastic cerebellar degeneration
RPE	Retinal pigment epithelium
VGCC	Voltage-gated calcium channel
