# Supplementary material for: Cancer-Associated Retinopathy Presenting with Paraneoplastic Cerebellar Degeneration in Tonsillar Squamous Cell Carcinoma: A Case Report
Source: Reports (MDPI). 2026 Sep 11;9(3):307. doi: 10.3390/reports9030307 (PMC13611654; doi:10.3390/reports9030307)
Supplement: Supplementary file 1 [file reports-09-00307-s001.zip › reports-4532423-supplementary.pdf]

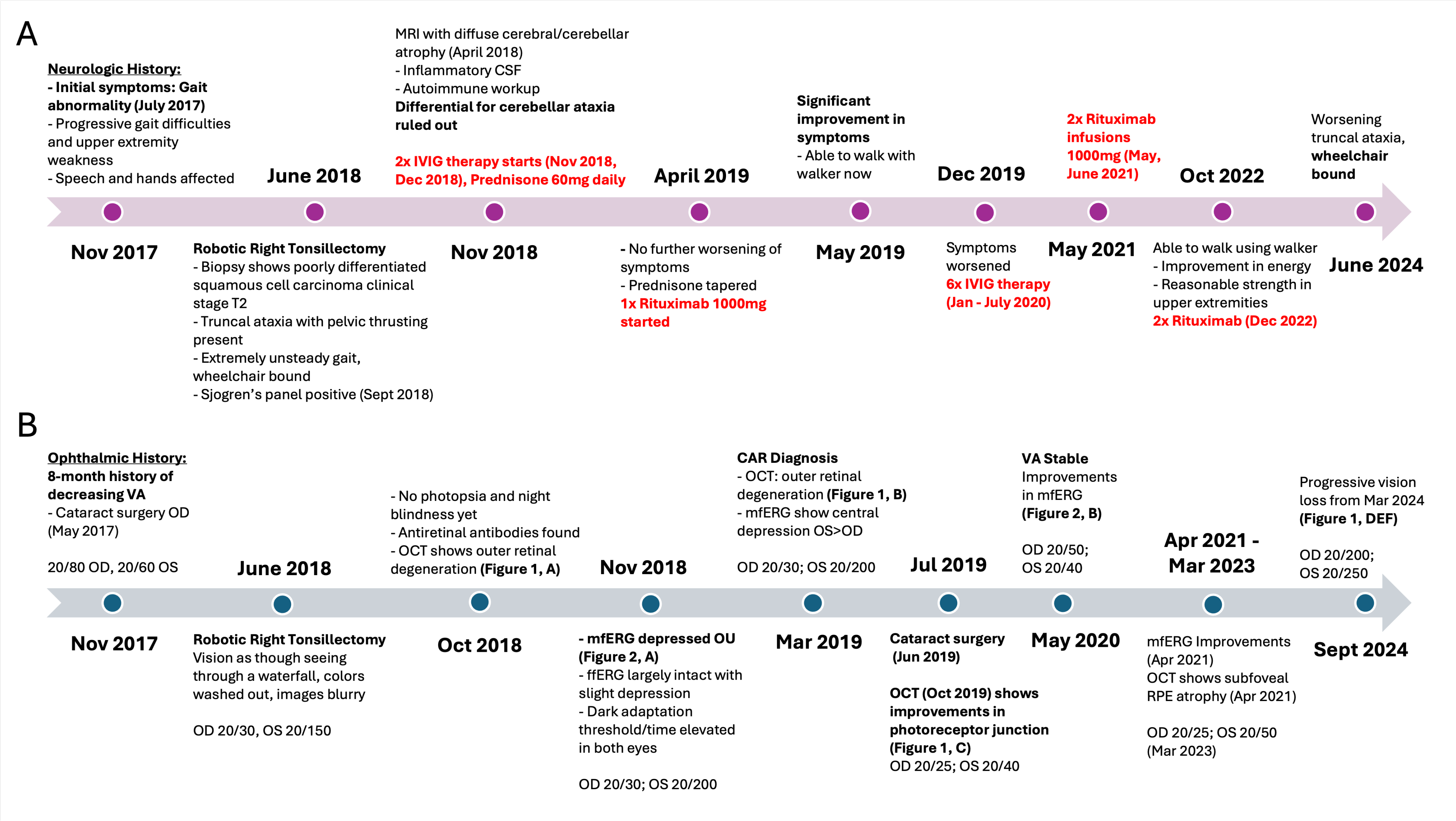
A
Neurologic History:
- Initial symptoms: Gait abnormality (July 2017)
- Progressive gait difficulties and upper extremity weakness
- Speech and hands affected
Nov 2017
June 2018
Robotic Right Tonsillectomy
- Biopsy shows poorly differentiated squamous cell carcinoma clinical stage T2
- Truncal ataxia with pelvic thrusting present
- Extremely unsteady gait, wheelchair bound
- Sjogren's panel positive (Sept 2018)
MRI with diffuse cerebral/cerebellar atrophy (April 2018)
- Inflammatory CSF
- Autoimmune workup
Differential for cerebellar ataxia ruled out
2x IVIG therapy starts (Nov 2018, Dec 2018), Prednisone 60mg daily
Nov 2018
April 2019
- No further worsening of symptoms
- Prednisone tapered
1x Rituximab 1000mg started
Significant improvement in symptoms
- Able to walk with walker now
May 2019
Dec 2019
Symptoms worsened
6x IVIG therapy (Jan - July 2020)
2x Rituximab infusions 1000mg (May, June 2021)
May 2021
Oct 2022
Able to walk using walker
- Improvement in energy
- Reasonable strength in upper extremities
2x Rituximab (Dec 2022)
Worsening truncal ataxia, wheelchair bound
June 2024
B
Ophthalmic History:
8-month history of decreasing VA
- Cataract surgery OD (May 2017)
20/80 OD, 20/60 OS
Nov 2017
June 2018
Robotic Right Tonsillectomy
Vision as though seeing through a waterfall, colors washed out, images blurry
OD 20/30, OS 20/150
- No photopsia and night blindness yet
- Antiretinal antibodies found
- OCT shows outer retinal degeneration (Figure 1, A)
Oct 2018
Nov 2018
- mfERG depressed OU (Figure 2, A)
- ffERG largely intact with slight depression
- Dark adaptation threshold/time elevated in both eyes
OD 20/30; OS 20/200
CAR Diagnosis
- OCT: outer retinal degeneration (Figure 1, B)
- mfERG show central depression OS>OD
OD 20/30; OS 20/200
Mar 2019
Jul 2019
Cataract surgery (Jun 2019)
OCT (Oct 2019) shows improvements in photoreceptor junction (Figure 1, C)
OD 20/25; OS 20/40
VA Stable
Improvements in mfERG (Figure 2, B)
OD 20/50; OS 20/40
May 2020
Apr 2021 - Mar 2023
mfERG Improvements (Apr 2021)
OCT shows subfoveal RPE atrophy (Apr 2021)
OD 20/25; OS 20/50 (Mar 2023)
Progressive vision loss from Mar 2024 (Figure 1, DEF)
OD 20/200; OS 20/250
Sept 2024
